# eggNOG-mapper v2: Functional Annotation, Orthology Assignments, and Domain Prediction at the Metagenomic Scale

**DOI:** 10.1093/molbev/msab293

**Published:** 2021-10-01

**Authors:** Carlos P Cantalapiedra, Ana Hernández-Plaza, Ivica Letunic, Peer Bork, Jaime Huerta-Cepas

**Affiliations:** 1 Centro de Biotecnologia y Genomica de Plantas, Universidad Politécnica de Madrid (UPM) – Instituto Nacional de Investigación y Tecnología Agraria y Alimentaria (INIA), Campus de Montegancedo-UPM, Madrid, Spain; 2 Biobyte Solutions GmbH, Heidelberg, Germany; 3 European Molecular Biology Laboratory, Structural and Computational Biology Unit, Heidelberg, Germany; 4 Department of Bioinformatics, Biocenter, University of Würzburg, Würzburg, Germany; 5 Yonsei Frontier Lab (YFL), Yonsei University, Seoul, South Korea

**Keywords:** metagenomics, functional annotation, computational genomics, bioinformatics

## Abstract

Even though automated functional annotation of genes represents a fundamental step in most genomic and metagenomic workflows, it remains challenging at large scales. Here, we describe a major upgrade to eggNOG-mapper, a tool for functional annotation based on precomputed orthology assignments, now optimized for vast (meta)genomic data sets. Improvements in version 2 include a full update of both the genomes and functional databases to those from eggNOG v5, as well as several efficiency enhancements and new features. Most notably, eggNOG-mapper v2 now allows for: 1) de novo gene prediction from raw contigs, 2) built-in pairwise orthology prediction, 3) fast protein domain discovery, and 4) automated GFF decoration. eggNOG-mapper v2 is available as a standalone tool or as an online service at http://eggnog-mapper.embl.de.

## Introduction

**Fig. 1:**
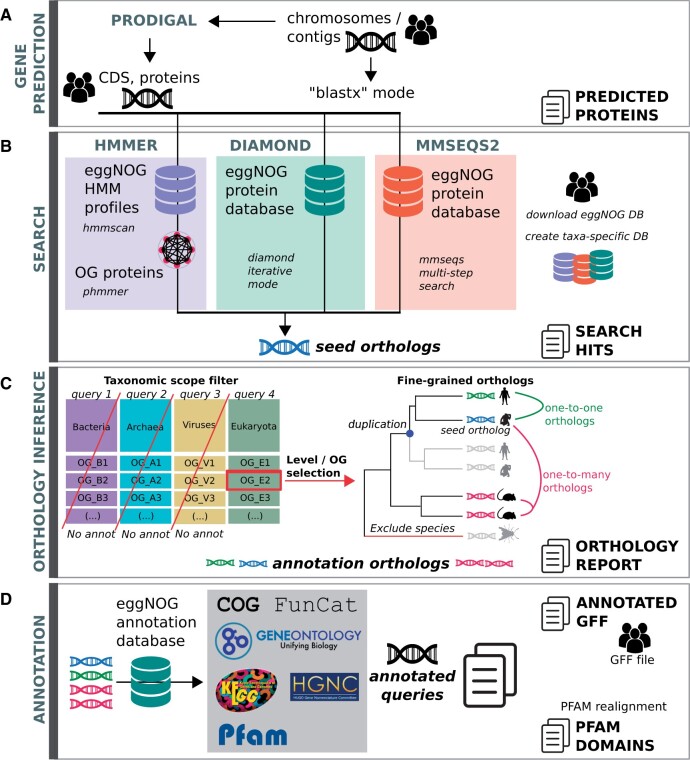
Workflow and new features of eggNOG-mapper v2. (*A*) The gene prediction stage uses Prodigal to perform protein prediction from assembled contigs. (*B*) During the search stage, HMMER3, Diamond, or MMseqs2 can be used to align the input proteins to eggNOG v5. (*C*) During the orthology inference stage, a report of orthologs is generated based on the desired taxonomic scope. (*D*) Finally, protein annotations and domains are transferred from orthologs to the queries and reported as tabular and GFF files.

Inference of gene function via orthology, rather than by homology detection, is generally considered the most reliable approach for transferring functional information between molecular sequences, as orthologs are expected to retain function more often than paralogs (Gabaldón and Koonin 2013; Glover et al. 2019). However, since delineating orthology is highly demanding (both computationally and algorithmically), most automated methods rely on homology-based annotations (Götz et al. 2008; Seemann 2014; Blum et al. 2021). EggNOG-mapper relies on the eggNOG database (Huerta-Cepas et al. 2019) of orthologs groups (OGs), covering thousands of bacterial, archaeal, and eukaryotic organisms. For this, it takes advantage of the precomputed phylogenies inferred for each OG to efficiently refine orthology assignments and therefore minimize the transferring of annotations from putative in-paralogs. The method was originally proven to provide more accurate predictions than homology-based approaches (Huerta-Cepas et al. 2017), while preserving computational performance at the genomic and metagenomic scale. Here, we present eggNOG-mapper v2, a major upgrade featuring improvements in annotation coverage, overall performance, and program capabilities (fig. 1).

## Coverage and Performance Improvements

**Fig. 2:**
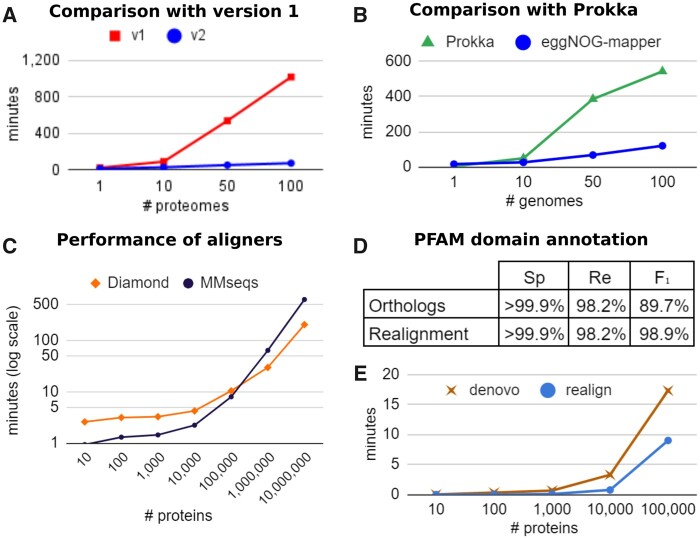
Performance of eggNOG-mapper v2. (*A*) average minutes to annotate input proteomes. EggNOG-mapper v2 (blue) against eggNOG-mapper v1 (red). (*B*) average minutes to annotate input genomes. EggNOG-mapper v2 (blue) against Prokka (green). (*C*) average minutes (in log scale) to annotate input proteins. MMseqs2 (-s 2,4,6; black) against Diamond (iterate/sensitive mode; orange). (*D*) Specificity (Sp), recall (Re), and F_1_ score, of PFAM domain annotation either from direct transference from orthologs, or after realignment. Full de novo realignment results were used as reference. (*E*) average minutes for PFAM domain annotation, using either PFAM full de novo (brown) or realign to orthologs domains (blue) modes. Benchmark setup: tests in (*A*) and (*B*) were done on 20 sets of 1–100 random proteomes (*A*) or genomes (*B*) from (Almeida et al. 2021), and executed using 10 CPUs and 80 GB of RAM. Tests in (*C*) were done on 35 random sets of 10–10,000,000 proteins from Progenomes v2 (Mende et al. 2020), using 30 CPUs and 240 GB of RAM. Tests in (*Dm*) and (*E*) as in (*C*), only for sets of 10–100,000 proteins.

The underlying genome database has been updated to be in sync with eggNOG v5, spanning 4.4 million OGs and more than twice the number of organisms than in the previous version. This improvement increases annotation coverage and phylogenetic resolution, particularly noticeable when analyzing large metagenomic data sets. For instance, the reannotation of 1.75 million proteins randomly subsampled from a human-gut metagenomic gene catalog (Almeida et al. 2021) yielded a 3.23% increase in annotation coverage (56,569 newly annotated proteins), compared with eggNOG-mapper version 1. The phylogenetic resolution was also improved, obtaining significantly better alignment scores for the query sequences than previous versions (Wilcoxon test W = 1.2E + 12, *P*-value < 2.2E − 16). Moreover, although the underlying databases have doubled in size, eggNOG-mapper v2 improves the annotation rate (annotated queries per second) by 16% on average, compared with previous versions. The most important changes regarding computational enhancements relate to database optimizations, allowing for faster queries and parallelization, and a new memory-based mode that significantly reduces the impact of slow I/O disk operations. Taken together, these changes improve annotation rates by 608% on average, with respect to eggNOG-mapper v1 (fig. 2*A*). Compared with Prokka (Seemann 2014), one of the fastest annotation tools available for prokaryotic genomes according to recent benchmarks (Shaffer et al. 2020), eggNOG-mapper runs faster, especially on large metagenomic data sets (fig. 2*B*).

## ORF Prediction

Another major capability added to the new eggNOG-mapper workflow is predicting ORFs directly from assembled contigs (fig. 1*A*). ORF detection, only available for prokaryotic assemblies, is performed using Prodigal (Hyatt et al. 2010), which provides the protein sequences to be used by eggNOG-mapper for functional annotation. Prodigal modes (“normal,” “anonymous,” and “training”) as well as custom translation tables can be further chosen by the user.

## Sequence Mapping Modes

Additionally, we have broadened the options for the initial sequence-mapping step carried out by eggNOG-mapper (fig. 1*B*). Now, Diamond, MMseqs2, and HMMER3 (Mistry et al. 2013) modes are available, each recommended for different use cases. The default Diamond mode provides the best balance between speed and memory consumption. EggNOG-mapper v2 also implements the new Diamond mode ‘–iterate’, which iteratively applies different levels of sensitivity. Using the new –iterate mode, Diamond is twice as fast as MMseqs2 for query sets of 1,000,000, and three times faster for 10,000,000 sequences. The MMseqs2 mode provides faster results than Diamond (fig. 2*C*) for query sets up to 100,000 sequences for comparable sensitivity. When input data are nucleotide sequences, a direct translation is done assuming they represent coding sequences starting in an open reading frame. Alternatively, both Diamond and MMseqs2 can be executed in blastx-like mode, particularly useful when using sequencing reads as input data. For the annotation of long, error-prone sequencing reads, the frameshift option of Diamond can also be enabled. The HMMER3 mode is significantly slower than the other two and requires heavy databases to be downloaded. However, HMM-based searches might aid in the annotation of sequences for which distant homology relationships against the eggNOG v5 OGs cannot be detected by Diamond and MMseqs modes.

## Adjusting Taxonomic Scopes

Another new feature now available with eggNOG-mapper v2 is the possibility of creating custom annotation databases constrained to specific taxonomic groups. For instance, users could easily create databases spanning only their domain or phylum of interest, therefore reducing computational times of subsequent annotation jobs. Moreover, the new version provides enhanced options to control the taxonomic scope (fig. 1*C*) used for transferring functional annotations, which can be adjusted from automatic mode (recommended for mixed metagenomic data sets) to lineage-specific scopes (preventing transferring functional terms from orthologs of unwanted lineages).

## Orthology Reports

Taking advantage of the rapid orthology assignments performed by eggNOG-mapper, it is now possible to report pairwise orthology relationships for each query against any of the genomes covered by eggNOG v5 (fig. 1*C*). Although this feature is not intended to substitute more precise orthology prediction methods, it provides a very quick and simple “first-pass” approach to obtain pairwise relationships between query sequences and all eggNOG v5 organisms. Orthology reports can be further adjusted by specifying the target taxa and the type of orthologs to be reported (i.e., one-to-one, many-to-many).

## Annotation Sources

In order to provide an integrated report of functional annotations per query, eggNOG-mapper v2 offers new annotation sources and improved reports (fig. 1*D*). The functional annotation sources, which provide different levels of coverage (supplementary fig. S1, Supplementary Material online), are: predicted protein name; KEGG pathways, modules, and orthologs (Kanehisa et al. 2017); Gene Ontology labels (Gene Ontology Consortium 2018); EC numbers, BiGG reactions (Norsigian et al. 2020); CAZy terms (Lombard et al. 2014); COG functional categories (Tatusov et al. 2000); eggNOG OGs; and free text descriptions at all taxonomic levels. Reports are generated in tab-delimited and/or XLSX file formats. Moreover, when ORF prediction mode is enabled, proteins used to annotate are reported in FASTA format, together with a functionally decorated GFF file. Alternatively, eggNOG-mapper annotation reports can be used to decorate any custom GFF file.

## Protein Domain Annotations

Along with the functional terms annotated per query, this new version of eggNOG-mapper provides PFAM (Mistry et al. 2021) and SMART (Letunic et al. 2021) protein domain predictions. PFAM domain annotations are by default transferred from the inferred orthologs with very little impact on computational cost, but also with a small proportion of false-positive and negative predictions (F1 score 89.7%, fig. 2*D*). Optionally, de novo PFAM domain annotation is also available at large scales, both as a refinement phase for the orthology-based predictions (thus keeping the computational cost very low, while eliminating the risk of false positives; *F*_1_ score 98.9%, fig. 2*D*) or by full computation (obtaining native results independent from orthology predictions). When using the de novo approach, HMMER3 searches are executed using in-memory mode for higher efficiency. Moreover, GA-based thresholds and PFAM clan disambiguation are automatically applied. Performance comparisons between the different modes are shown in figure 2*E.*

## Features Comparison with Other Functional Annotation Tools

Although accuracy and performance are the main goal of eggNOG-mapper v2, other practical differences might motivate its use, or not, compared with alternative software. Those practical issues include the type of input and output data, the functional sources used to annotate query sequences, and the target taxonomic scope of each program.

EggNOG-mapper is tightly bound to the eggNOG database, which covers a wide range of prokaryotic and eukaryotic organisms, and provides normalized functional annotations from multiple sources (see previous sections). As eggNOG is based on a curated selection of representative species spanning the whole tree of life, it provides a broad annotation coverage while keeping redundancy low. Other tools use smaller reference databases (e.g., SwissProt [UniProt Consortium 2021]) for general functional annotations but incorporate niche-specific functional databases for specific purposes. For instance, Prokka is suited for the annotation for prokaryotic-only genomes and includes the detection of tRNAs, transposases and signal peptides (which are not specifically targeted by eggNOG-mapper). DRAM (Shaffer et al. 2020) and MicrobiomeAnnotator (Ruiz-Perez et al. 2021) use broad annotation sources for microbial data sets such as KOFam/KEGG classification. Both provide optional mappings to large protein reference databases comparable to eggNOG (e.g., trEMBL or Uniref90), but at the cost of more computational resources and time (Ruiz-Perez et al. 2021). In the case of DRAM, additional genomic features can be annotated, including rRNAs, tRNAs, peptidases, and carbohydrate-active enzymes. Mantis (Queirós et al. 2021) and InterProScan allow users to annotate sequences based on multiple HMM-based database sources, including eggNOG, PFAM, or PANTHER (Mi et al. 2010).

On the other hand, this new version of eggNOG-mapper is particularly focused on improving the annotation process at the metagenomic scale. For instance, it incorporates the possibility of using Prodigal for the automatic prediction of open reading frames (ORFs) out of raw contigs. Those options are not commonly available in other annotation tools, with only Prokka and DRAM providing similar capabilities. Moreover, eggNOG-mapper v2 allows for the direct annotation of reads via blastx-like searches, which is not a recommended mode in terms of accuracy, but it offers the possibility of obtaining functional profiles of metagenomic samples before assembling.

Regarding outputs, eggNOG-mapper is optimized for large-scale annotation jobs, producing GFF files decorated with functional annotations, tab-delimited files with the predicted functional terms per query, orthology assignment tables and taxonomic placements. Other tools provide similar output files (except for the orthology and taxonomic predictions), with only DRAM and MicrobiomeAnnotator providing further statistics and graphical outputs summarizing the results. Notably, Prokka can be set to produce Genbank and Sequin files compliant with the Genbank/ENA/DDJB format, thus facilitating the uploading of annotated genomes to public databases.

## Conclusions

Overall, eggNOG-mapper v2 provides a more efficient, versatile, and scalable automated functional annotation workflow than its predecessor. Standalone versions are available at GitHub (https://github.com/eggnogdb/eggnog-mapper), together with extensive documentation and usage examples (https://github.com/eggnogdb/eggnog-mapper/wiki). For convenience, an online service for the annotation of large genomic and metagenomic data sets is also available at http://eggnog-mapper.embl.de.

## Supplementary Material

Supplementary data are available at *Molecular Biology and Evolution* online.

## Supplementary Material

msab293_Supplementary_Data
