# Supplementary material for: eggNOG-mapper v2: Functional Annotation, Orthology Assignments, and Domain Prediction at the Metagenomic Scale
Source: Mol Biol Evol. 2021 Oct 1;38(12):5825–9. doi: 10.1093/molbev/msab293 (PMC8662613; doi:10.1093/molbev/msab293)
Supplement: msab293_Supplementary_Data [file msab293_supplementary_data.pdf]

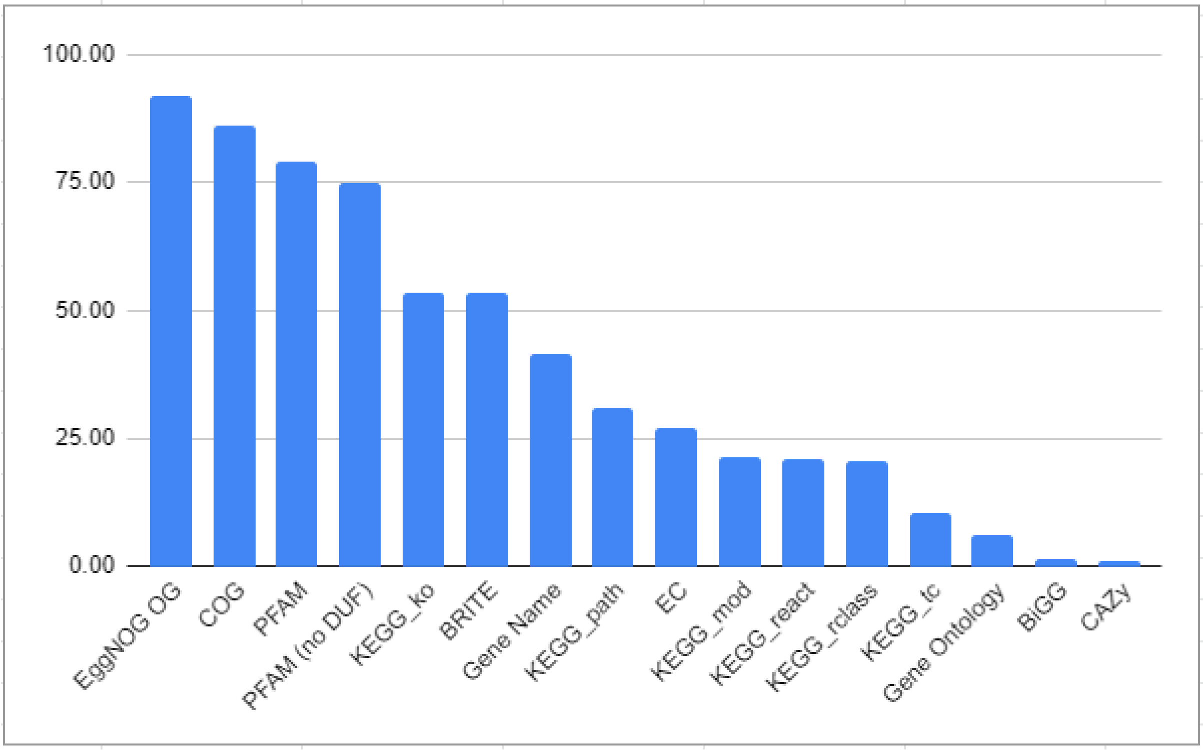

Supplementary Figure 1. Functional annotation sources included in eggNOG-mapper v2, arranged by the average annotation coverage (% of annotated proteins) in a test with 25 random sets of between 10 and 100,000 proteins from the Progenomes database. PFAM domain annotation obtained from realignment of queries to orthologs domains ('--pfam_realign realign' option). The "PFAM (no DUF)" category includes only proteins annotated with at least one non-"DUF" domain.
